# Supplementary material for: Patterns of Intron Gain and Loss in Fungi
Source: PLoS Biol. 2004 Nov 30;2(12):e422. doi: 10.1371/journal.pbio.0020422 (PMC532390; doi:10.1371/journal.pbio.0020422)
Supplement: Table S1 — Also available at http://genes.mit.edu/NielsenEtAl/. (4.3 MB ZIP). [file pbio.0020422.st001.zip › NielsenEtAl/html/366.html]

AN1778.1.NCU10048.1.MG00599.1.FG09312.1


```
 CLUSTAL W (1.82) Multiple Sequence Alignments - Introns Inserted


Sequence 1: NCU10048.1	309 aa
Sequence 2: FG09312.1	467 aa
Sequence 3: MG00599.1	373 aa
Sequence 4: AN1778.1	349 aa
Alignment Length: 491 aa
Number Identitical Residues: 182 aa
Alignment Score (without introns) 8303


MG00599.1 	MHPPAATTALARSCASVASTLVRGRAINLPRTQQPCRTLSSIAVSPARPSNHPDQVSTAA
NCU10048.1	------------------------------------------------------------
FG09312.1 	-----------------MSSLIN------PTMRSCTSFLSRMPVSSYRPAIR-SLGSSVR
AN1778.1  	------------------MTFLR------IATKRAIYLHRPANPALPTSSILPVLHSTNV
          	                   :                        :   .:  .   ::  

MG00599.1 	SQKPQQQQQCRHSSHSPMGAPIANQRKKVTLGTLRSLYKKGEPITVMTAHDFPSAHVADA
NCU10048.1	-----------------MGLPPTNPRKKVTLNTLQSMYKKGEKITMITAHDFPSAHVAEH
FG09312.1 	PLIIRGVAQSRYSSHSPMGAPPAAQRKKVTVGTLRSMHRKGDPITVMTAHDFPSGHVADH
AN1778.1  	ATRVPSPCAIRHSSHSPLGAAQANPRKKVTMQTLRNLYKKGEPITMLTAHDFPSAHVADA
          	.           :: :.:* . :  *****: **:.:::**: **::*******.***: 

MG00599.1 	AGMDIILVGDSLAMVALGMEDTSEVVLDEMLLHCRSVARATKSAFT0IGDLPMGSYEVSP
NCU10048.1	AGMDMILVGDSLAMVALGMEDTSEVLVEEMLLHCRSVARATSRAFT0VGDLPMGSYEISP
FG09312.1 	AGMDIILVGDSLAMVALGMEDTSEVIMEEMLLHCRSVARATKSAFT0VGDLPMGTYEISP
AN1778.1  	AGMDMILVGDSLAMVALGMQDTSEVTLDDMLVHCRSVARAAQSAFT0VSDLPMGSYEVSP
          	****:**************:***** :::**:********:. *** :.*****:**:**

MG00599.1 	EQALQTAIRFVKEGRVQGIKIEGGREMAPTIRKITGAGIPVLGHIGLTPQRQHSLGGFRV
NCU10048.1	QQALSTAIRFIKEGRVSAVKLEGGAEMAPTIRAITTAGIPVLAHIGLTPQRQNALGGFRV
FG09312.1 	DQALATAIRFIKEGRVQGVKLEGGKEMAPTIKKITTAGIPVLGHIGLTPQRQNALGGFRV
AN1778.1  	EQALQSAIRIVKEGRVQGVKLEGGEEMAPAIKRITTAGIPVVGHIGLTPQRQNALGGFRV
          	:*** :***::*****..:*:*** ****:*: ** *****:.*********::******

MG00599.1 	QGKTSAAAQSLLQDALAVQEAGCFATVVEAVPAEVSALLTKKLSIPTIGIGAGNGCSGQV
NCU10048.1	QGKSKESAKRLLRDALAVQEAGAFAVVVEAVPQEVAAFITGLLKIPTIGIGAGNGTSGQV
FG09312.1 	QGKTSSGAMSILEDALALQEAGCFAMVVEAVPAEVAALITEKLSVPTIGIGAGNGTSGQV
AN1778.1  	QGKSTTDALKLLKDALAVQEAGAFMIVIEAVPPEIASIVTQKLSVPTIGIGAGNGCSGQV
          	***:.  *  :*.****:****.*  *:**** *:::::*  *.:********** ****

MG00599.1 	LVQIDMTGNFPPGRFLPKFVKKYGDVWGESMRAIEAYRDEVKSRAYPGPEHTYPMPSEEL
NCU10048.1	LVQADMTGNFPPGRFLPKFVKKYGDVWGEALRAIETYRDEVKGGSYPAEEHTYPMAKEEV
FG09312.1 	LVQVDMIGNFPPGRFLPKFVKKYGDVWGESLRAIEAYRDDVKSRQYPGPEHTYPISAEEL
AN1778.1  	LVQIDMTGNFPPGRFLPKFVKQYANVWNEALQGIQQYREEVKSRAYPAEQHTYPIPKEEL
          	*** ** **************:*.:**.*:::.*: **::**.  **. :****:. **:

MG00599.1 	AAFKA-~------------------------~----------------------------
NCU10048.1	EAFVG-~------------------------~----------------------------
FG09312.1 	ESFTKA0IEEIDLVGLIQKQVLESKQQQVDR0TLSVSLHLNFFLKYLTTSSKRLVALILH
AN1778.1  	VEFQK-~------------------------~----------------------------
          	  *                                                         

MG00599.1 	-----------------------VIEGEKSLE--------------~-------------
NCU10048.1	-------------------DVRRLMNANASAKAEG---EGEKDA--~-------------
FG09312.1 	TSWANNGADPTGLNRTTILSVLKLPAASSAAVASLGRSASTTDADM0IQGFSVFQFGGNM
AN1778.1  	-------------------------AVDELPEEK------------~-------------
          	                           .     .                          

MG00599.1 	----~-----------
NCU10048.1	----~-----------
FG09312.1 	FVGE1SYVFIMTPQAF
AN1778.1  	----~-----------
          	
```
